# Bioproduction of High-Concentration 4-Vinylguaiacol Using Whole-Cell Catalysis Harboring an Organic Solvent-Tolerant Phenolic Acid Decarboxylase From *Bacillus atrophaeus*

**DOI:** 10.3389/fmicb.2019.01798

**Published:** 2019-08-06

**Authors:** Lulu Li, Liangkun Long, Shaojun Ding

**Affiliations:** The Co-innovation Center of Efficient Processing and Utilization of Forest Resources, Jiangsu Key Lab for the Chemistry & Utilization of Agricultural and Forest Biomass, College of Chemical Engineering, Nanjing Forestry University, Nanjing, China

**Keywords:** phenolic acid decarboxylase, ferulic acid, 4-vinylguaiacol, biphasic biotransformation system, whole-cell catalysis

## Abstract

The compound 4-vinyl guaiacol (4-VG) is highly valued and widely applied in the pharmaceutical, cosmetic, and food industries. The bioproduction of 4-VG from ferulic acid (FA) by non-oxidative decarboxylation using phenolic acid decarboxylases is promising but has been hampered by low conversion yields and final product concentrations due to the toxicities of 4-VG and FA. In the current study, a new phenolic acid decarboxylase (BaPAD) was characterized from *Bacillus atrophaeus*. The BaPAD possessed excellent catalytic activity and stability in various organic solvents. Whole *Escherichia coli* cells harboring intracellular BaPAD exhibited greater tolerances to FA and 4-VG than those of free BaPAD. A highly efficient aqueous-organic biphasic system was established using 1-octanol as the optimal organic phase for whole-cell catalysis. In this system, a very high concentration (1580 mM, 237.3 g/L) of 4-VG was achieved in a 2 L working volume bioreactor, and the molar conversion yield and productivity reached 98.9% and 18.3 g/L/h in 13 h, respectively.

## Introduction

The bioproduction of fine flavors and fragrances has expanded rapidly in recent years (Paz et al., 2016; Sales et al., 2018). Among these aromatic compounds, 4-vinyl guaiacol (4-VG), also known as 3-methoxy 4-hydroxystyrene, is a particularly valuable product in the food, cosmetic, pharmaceutical, and chemical industries (Matte et al., 2010; Peña et al., 2010; Sun et al., 2018). This phenol metabolite with a spicy clove-like aroma can be obtained from the decarboxylation of ferulic acid (FA), a phenolic compound found widely in lignocellulosic materials and is particularly rich in agricultural by-products, such as corn, rice and wheat bran, and corn hulls (Mattila and Hellström, 2007; Wong et al., 2011; Jiang et al., 2016). The commercial price of 4-VG is forty times greater than that of FA (Landete et al., 2010). The global demand of 4-VG has been steadily increasing due to its growing number of applications in many fields (Baqueiro-Pena et al., 2010; Mishra et al., 2014b; Tinikul et al., 2018). Currently, commercial 4-VG is obtained by chemical decarboxylation of FA using metal catalysts under harsh conditions, which raises concerns about the product safety and hazardous wastes (Dickstein et al., 2007). Therefore, the use of microorganisms or enzymes as biocatalysts is a promising method for the production of 4-VG under mild processing conditions. Although the use of some yeasts (Donaghy et al., 1999; Mathew et al., 2007) and fungi (Topakas et al., 2003) has been evaluated previously, bacteria are the most widely assayed microorganisms, including *Streptomyces setonii* (Max et al., 2012; Salgado et al., 2012a), *Cupriavidus* sp. (Chai et al., 2013), *Enterobacter* sp. (Li et al., 2008), *Lactobacillus farciminis* (Hadiza et al., 2012), and *Bacillus* spp. (Karmakar et al., 2000; Paz et al., 2016). From these studies, the yield of 4-VG was found to be unsatisfactory due to the low catalytic activities of wild microorganisms. For instance, Max et al. (2012) reported that 885.1 mg/L (5.7 mM) of 4-VG was obtained by the decarboxylation of FA using a strain of *S. setonii* ATCC 39116, and similar amounts (720 mg/L-760 mg/L, 4.8 mM-5.1 mM) of 4-VG were produced from FA through biotransformations using different bacterial species (Hua et al., 2013; Sun et al., 2018).

To promote the efficiency of transforming FA to 4-VG, the recombinant phenolic acid decarboxylases cloned from various microorganisms, such as *Enterobacter* sp. (Gu et al., 2011), *Bacillus* spp. (Prim et al., 2003; Mishra et al., 2014a), and *Aspergillus luchuensis* (Mayumi et al., 2018), were used as biocatalysts for the bioproduction of 4-VG. However, the toxicities of 4-VG and FA impeded the achievement of higher titers in the biocatalytic process, and the final product concentration was still significantly lower than the minimum required to feed a conventional downstream process. This problem can be alleviated by the implementation of a two-phase system with an organic solvent as the second liquid phase (Heipieper et al., 2007). A typical two-phase biotransformation system (TPBS) consists of an organic phase and an aqueous phase containing biocatalysts and media. TPBSs have been used as platforms for environmental pollutant treatment, fermentation, and biocatalysis (Malinowski, 2001; Heipieper et al., 2007; Salgado et al., 2014). The biphasic system enables the 4-VG to be selectively transferred into the organic phase, while the FA and biocatalysts remain in the aqueous phase, thereby minimizing the toxicity of 4-VG and enhancing the final product concentration, conversion yield, and product recovery (Yang et al., 2009; Jung et al., 2013). However, the use of a biphasic system requires the enzyme or microorganism to have a good tolerance to organic solvents and retain a high activity in the presence of organic solvents. Hu et al. (2015) used an organic-solvent-tolerant phenolic acid decarboxylase (BLPAD) cloned from *Bacillus licheniformis* to convert 500 mM FA to 354.5 mM (58.3 g/L) 4-VG at a molar conversion yield of 70.9% in an organic–aqueous biphasic system. Based on this research, Chen et al. (2018) employed a fed-batch process to achieve high cell density cultivation of recombinant *Escherichia coli* expressing phenolic acid decarboxylase and realized large-scale 4-VG bioproduction in a 5 L bioreactor using a biphasic emulsion system with a substrate feeding strategy. The activity of the BLPAD in the recombinant *E. coli* reached up to 531 U/mL, and the final concentration of 4-VG increased to 865.2 mM (129.9 g/L), corresponding to a molar conversion yield of 85.6%.

In this study, we identified a phenolic acid decarboxylase (BaPAD) from *Bacillus atrophaeus* by genome sequence mining. The BaPAD was heterologously overexpressed as a soluble protein in *E. coli*. The BaPAD exhibited a relatively high stability over a wide range of pH values and high temperatures. The BaPAD exhibited an excellent catalytic activity and good tolerance to various organic solvents. An organic solvent/aqueous biphasic system containing 1-octanol and an equal volume of aqueous phase was established by comprehensively evaluating the toxicity of 4-VG, FA, and various organic solvents to the recombinant enzyme and *E. coli* cells expressing the BaPAD. In this system, a large amount (approximately 310 g/L) of FA could be almost completely converted to 4-VG in a 5 L bioreactor, which is the highest value reported to date. This work clearly demonstrated the feasibility of scaling up 4-VG bioproduction using whole-cell catalysis.

## Materials and Methods

### Culture Medium and Chemicals

Luria–Bertani (LB) medium was used for cell cultivation of *E. coli*. Hydroxycinnamic acids and the corresponding 4-vinylphenol derivatives, including FA, *p*-coumaric acid, caffeic acid, 4-vinylguaiacol, and 4-vinylphenol, were purchased from Sigma–Aldrich (St. Louis, MO, United States). Isopropyl-β-D thiogalactopyranoside (IPTG) was also obtained from Sigma–Aldrich. Yeast extract and tryptone were purchased from Oxoid (Basingstoke, England). Kanamycin was provided by Amresco (Solon, OH, United States). All the organic solvents used for whole-cell catalysis and high-performance liquid chromatography (HPLC) were of HPLC grade. The other chemicals used in this study were of analytical grade and were commercially available.

### Expression and Purification of BaPAD in *E. coli* BL21(DE3)

A codon-optimized phenolic acid decarboxylase gene (*bapad*, Genbank accession no. MN011580) based on the sequence (Genbank accession no. AKL86192.1) from *B. atrophaeus* was synthesized by Spring (Nanjing, China), and cloned into the vector pET28a (Novagen, Copenhagen, Denmark) at the restrictive sites of *Nco*I/*Xho*I to generate a pET28a-bapad vector. The recombinant pET28a-bapad vector was transformed into *E. coli* BL21(DE3) (Novagen, Germany), and BaPAD was overexpressed as a soluble protein in *E. coli* BL21(DE3), which was induced by 0.4 mM IPTG at 28°C for 12 h. Cells were collected by centrifugation at 8,000 rpm for 10 min. Cell pellets were used for enzyme purification or for whole cell catalysis. For enzyme purification, the cells were washed and resuspended in lysis buffer (50 mM NaH_2_PO_4_, 300 mM NaCl, pH 8.0). The cells were disrupted through ultrasonication, and the cell-free extract was collected by centrifugation at 10,000 rpm and 4°C for 10 min. The crude enzymes were purified by affinity chromatography using Ni–NTA Agarose gel (Qiagen, Valencia, CA, United States) according to the manufacturer’s manual. The enzyme purity and molecular weight of the purified BaPAD were estimated using sodium dodecyl sulfate-polyacrylamide gel electrophoresis (SDS- PAGE) [12.5% (w/v)]. The purified protein was stored at 4°C and used as soon as possible after purification.

### Enzymatic Properties of BaPAD

The protein concentration was determined by the Bradford assay (Bradford, 1976) using the Pierce BCA protein assay kit (Thermo scientific, Rockford, United States) with bovine serum albumin as the standard. The enzyme activity was determined in 1 mL reaction mixture containing 2 μg of purified BaPAD, 5 mM FA, and a 200 mM Na_2_HPO_4_-citric acid buffer (pH 5.5). The reaction was initiated at the optimal temperature (50°C) by adding enzyme to the reaction mixture. Two volumes of methanol were added to stop the reaction after 5 min. The amounts of remaining FA and generated 4-VG in the reaction mixture were determined by HPLC according to previously described (Hu et al., 2015). The kinetic parameters were determined by measuring the initial reaction velocity at different concentrations of *p*-coumaric acid, FA, and caffeic acid. The *K*_m_ and *V*_*max*_ values were determined by fitting the data to the Michaelis-Menten equation using the Graphpad Prism 7 non-linear regression program.

The effects of pH and temperature on the activity of the purified enzyme were investigated under the standard assay conditions described above in the pH and temperature ranges of 4.0–8.0 (200 mM Na_2_HPO_4_–citric acid buffer) and 20–70°C, respectively. The thermal stability was determined by measuring the residual activity after pre-incubating the enzyme at 45–55°C without a substrate for 0–100 min. The pH stability was estimated by measuring the residual activity after incubating the enzyme in a universal buffer (50 mM H_3_PO_4_, 50 mM CH_3_COOH, and 50 mM H_3_BO_3_ with pH values from 3.0 to 10.0 adjusted using 0.2 M NaOH) at room temperature for 1 h. The effects of metal ions, SDS, EDTA, and Triton-X on the BaPAD activity were determined in the presence of 5 and 10 mM concentrations of various metal ions, SDS, EDTA, and Triton-X under the standard assay conditions.

### Optimal pH and Temperature for Whole-Cell Catalysis

The optimal temperature and pH for the whole-cell catalysis were determined in 100 mL Teflon seal-cap bottles. The mixture consisted of 15 mL of a 200 mM Na_2_HPO_4_-citric acid buffer containing a 50 mM sodium ferulate solution and a specified number of cells (OD_600_ = 0.1). The reaction was performed in a shaker at 150 rpm for 1 h. The temperatures were 25, 28, 30, 35, and 37°C, and the pH ranged from 4.5 to 8.0. The concentrations of the generated 4-VG were detected by HPLC after dilution with methanol.

### The Influence of Organic Solvents on Activity and Stability of BaPAD

The influence of organic solvents on the BaPAD activity was examined by measuring the activity under the standard assay conditions in the presence of 50% (v/v) concentrations of various organic solvents with constant shaking (200 rpm) at 50°C for 20 min. To evaluate the BaPAD stability to various organic solvents, the BaPAD was incubated with 20, 30, and 50% (v/v) concentrations of various organic solvents under constant shaking at 200 rpm for 1 and 12 h, after which the residual activity of the BaPAD was measured under the standard assay conditions.

### The Molecular Toxicity Analysis of BaPAD and Whole Cells

To determine the toxicity of the substrate or product to the activity of the BaPAD and whole cells, the catalytic activity assay was measured under the standard assay conditions in the presence of different concentrations of FA (5, 10, 25, 50, 100, 200, 300, 400, and 500 mM) or 4-VG (1, 5, 10, 20, 30, 40, 50, and 75 mM) with BaPAD (10 μg) or whole cells (with the equivalent of 10 μg of the BaPAD protein) at 50°C and 30°C for 5 min, respectively.

To determine the combined toxicity of the residual organic solvents and 4-VG dissolved in the aqueous phase on the catalytic activity of the BaPAD and whole cells, a Na_2_HPO_4_-citric acid buffer (200 mM) was pre-saturated with 10 g/L (66.7 mM) or 100 g/L (666.7 mM) of 4-VG and equal volumes of different organic solvents at 37°C and 200 rpm for 48 h. Subsequently, the organic solvent was removed, and the saturated buffer was collected after centrifugation at 6500 rpm for 10 min. The activities of the BaPAD and whole cells were determined by using the saturated buffer under the standard assay conditions, as above described. The relative activity was calculated by defining the activity in the buffer prior to pre-saturating it with 4-VG and organic solvents to be 100%.

### Solvent Screening for Whole-Cell Catalysis in Biphasic System

To screen the favorable organic solvents for the biphasic reaction, whole-cell catalysis was carried out in the biphasic system using different organic solvents for 2 and 6 h. The concentration of FA in the Na_2_HPO_4_-citric acid buffer was increased to 200 mM to ensure the biphasic system with the selected organic solvent could work well at high concentrations of FA. The concentrations of residual FA and generated 4-VG were determined by HPLC.

### Bioproduction of 4-VG by Whole-Cell Catalysis in Bioreactor

The whole-cell catalysis for the bioproduction of 4-VG was performed in a 5 L bioreactor (Sartorius BIOSTAT^®^ Aplus, Goettingen, Germany) in a aqueous phase (200 mM, pH 6.5 Na_2_HPO_4_-citric acid buffer) or biphasic system comprising 1 L of aqueous phase and 1 L of 1-octanol. In both systems, aqueous phase initially contains 100 mM (for aqueous phase system) or 200 mM of FA (for biphasic system) and 7 g (wet weight) of whole cells at 30°C with an agitation speed of 120 rpm. FA was intermittently fed into the reaction mixture once the pH increased to 6.6 due to the consumption of FA, after which the feed was stopped until the pH declined to 6.4. Thus, the pH in the aqueous phase was maintained in a range of 6.5 ± 0.1 during the whole-cell catalysis process. All the reported data are averages of the duplicate experiments.

### Analytical Methods

Cell growth was monitored by direct measurements of the optical density (OD_600_) using a pure culture fluid as a blank control. FA and 4-VG in the organic and aqueous phases were quantified by HPLC (Agilent 1260 equipped with a quaternary pump and DAD detector) using a ZORBAX Eclipse Plus C18 (4.6 mm × 100 mm, Agilent, Palo Alto, CA, United States) column, as previously described for BLPAD (Hu et al., 2015). Quantitative analysis was performed using the external standard method. The retention times for FA and 4-VG were 6.8 and 21.8 min, respectively.

## Results

### Expression and Purification of BaPAD

**FIGURE 1:**
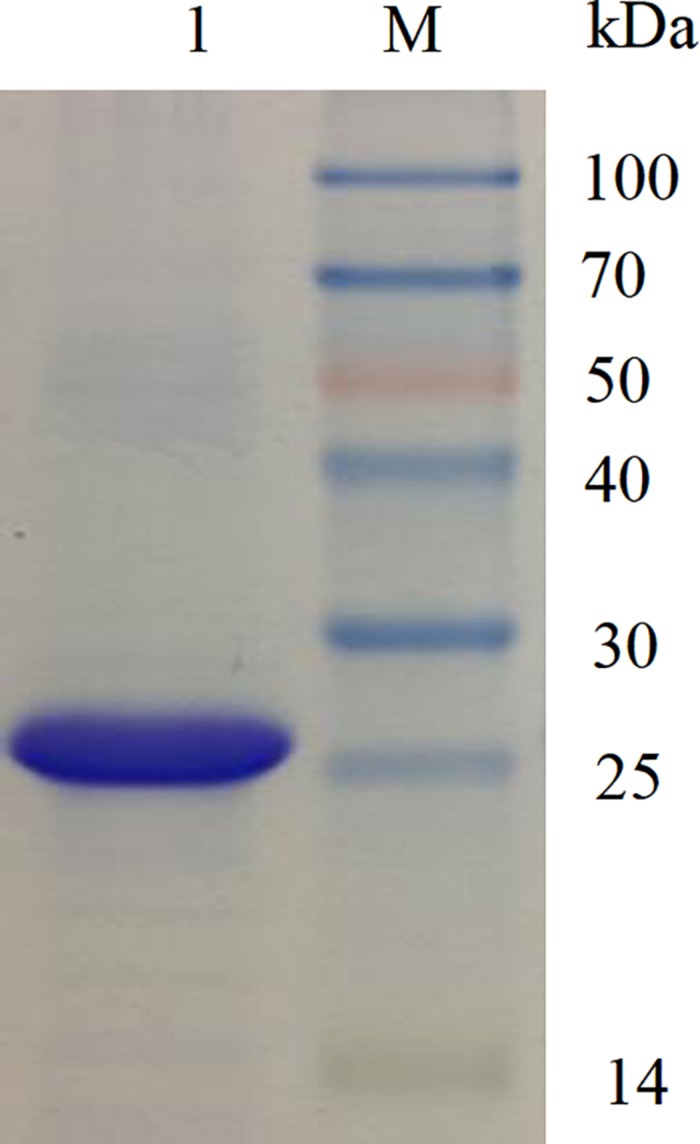
SDS-PAGE analysis of the purified BaPAD. Lane M: molecular weight of standard protein marker. Lane 1: the purified BaPAD.

The recombinant phenolic acid decarboxylase (BaPAD) from *B. atrophaeus* was overexpressed in the heterologous host *E. coli* using the pET28a^+^ expression vector. After induction under the optimal conditions (28°C and 400 μM IPTG) for 12 h, the BaPAD activity in *E. coli* reached up to 138.9 IU mL^–1^ (Supplementary Figure S1). The recombinant BaPAD with a 6 × His-tag at its N-terminus was affinity-purified using a Ni-NTA gel column. The purified BaPAD exhibited a single band with a molecular weight of about 25 kDa in the SDS-PAGE analysis (Figure 1).

### Characterization of Recombinant BaPAD

**FIGURE 2:**
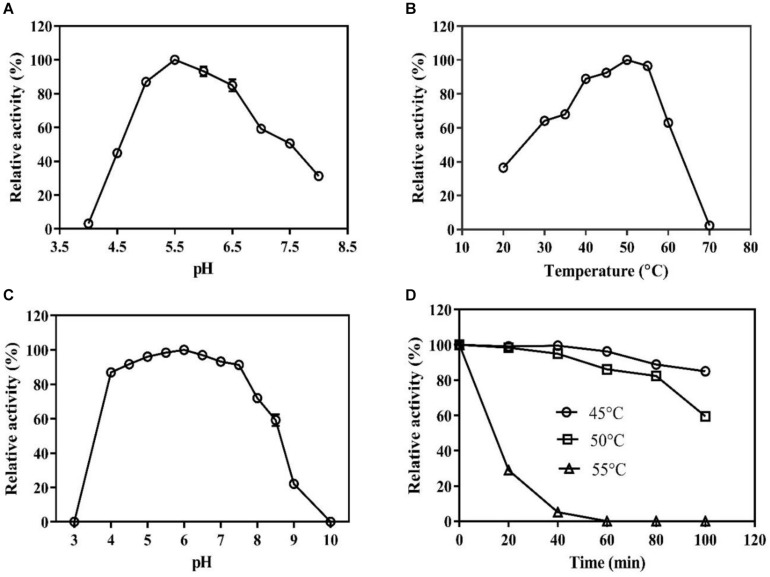
Biochemical characterization of BaPAD. **(A)** Effect of pH on the BaPAD activity using FA as a substrate at temperature 50°C. **(B)** Effect of temperature on the BaPAD activity at the optimum pH. **(C,D)** pH and temperature stability of BaPAD, respectively. 45°C (open circles), 50°C (open squares), and 55°C (open triangles).

The pH and temperature optima for the BaPAD activity were 5.5 and 50°C, respectively (Figures 2A,B). The BaPAD also exhibited over 80% relative activity in the pH range of 5.0–7.0 and over 60% between 30 and 60°C. The enzyme exhibited good stability in the pH range of 4.0–8.0 and retained over 70% of its initial activity (Figure 2C). The BaPAD was very stable at 45°C, and more than 90% of its initial activity was reserved after 100 min incubation. Moreover, approximately 60% of its initial activity was retained after 100 min incubation at 50°C, although the enzyme activity sharply declined at temperatures over 55°C (Figure 2D).

Cu^2+^ inhibited the BaPAD activity strongly at a concentration 10 mM, while other metal ions had only slight inhibitory effects (Supplementary Table S1). Additionally, the BaPAD activity was weakly inhibited by EDTA and Triton X-100, while SDS inhibited the enzyme activity completely (Supplementary Table S1), similar to the findings for FADase from *Enterobacter* sp. Px6-4 (Gu et al., 2011).

The substrate specificities of the purified BaPAD toward various phenolic acids (5 mM each) were examined under the standard assay conditions. The kinetic constant *K*_m_ values against *p*-coumaric, ferulic, and caffeic acids were 3.45 ± 1.28, 2.57 ± 2.21, and 1.68 ± 1.86 mM, and the *V*_*max*_ values were 775.90 ± 0.87, 440.61 ± 0.56, and 173.63 ± 2.31 U mg^–1^, respectively (Table 1). Among these compounds, BaPAD tended to favor *p*-coumaric over FA and caffeic acid as substrates. The relative ratio of the specific activities of BaPAD was approximately 100:70.5:31.7 toward *p*-coumaric acid, FA, and caffeic acid, as shown in Table 2.

**TABLE 1:** Kinetic parameters of BaPAD.

**Substrate**	**Kinetic parameter^a^**			
	***V*_*max*_ (IU/mg)**	***K*_m_ (mM)**	***k*_cat_ (s^–1^)**	***k*_cat_/*K*_m_^b^ (mM^–1^S^–1^)**
*p*-Coumaric acid	775.90 ± 0.87	3.45 ± 1.28	131.23 ± 2.38	38.05 ± 1.35
Ferulic acid	440.61 ± 0.56	2.57 ± 2.21	77.12 ± 1.56	29.30 ± 1.59
Caffeic acid	173.63 ± 2.31	1.68 ± 1.86	32.52 ± 0.89	19.30 ± 2.15

*^*a*^The values are the means of triplicate experiments with ± standard error (SE). ^*b*^The values were calculated assuming the native molecular mass as 21 kDa.*

**TABLE 2:** Substrate specificity of BaPAD.

**Substrate**	**Specific activity^a^ (IU/mg)**	**relative ratios**
*p*-Coumaric acid	253 ± 12.33	100
Ferulic acid	178.32 ± 0.55	70.48
Caffeic acid	80.2 ± 5.72	31.7

*^*a*^The values are the means of triplicate experiments with ± standard error (SE).*

### The Influence of Organic Solvents on Activity and Stability of BaPAD

The influence of organic solvents on the activity of BaPAD was determined by measuring the activity using FA in the presence of various organic solvents. As shown in Table 3, the BaPAD activity was activated by all the tested organic solvents except for petroleum ether (16% inhibition). *n*-Heptane showed the highest activation effect on the BaPAD activity, followed by *n*-hexane, toluene, *n*-decane, and *n*-octane. Unlike BLPAD (Hu et al., 2015), 1-octanol did not inhibit but activated the BaPAD activity. As shown in Table 4, BaPAD retained 90% or more of its initial activity after incubating the BaPAD with various organic solvents for 1 h at 37°C. More than 80% of its initial activity was retained after incubating the BaPAD with most of the tested organic solvents for 12 h at 37°C. Even though BaPAD was less stable in 1-octanol, approximately 57 to 80% of its initial activity was retained after incubating the BaPAD with different concentrations of 1-octanol for 12 h. These observations revealed that the BaPAD from *B. atrophaeus* possessed extensive organic solvent tolerance.

**TABLE 3:** Effects of different organic solvents on the recombinant BaPAD activity.

**Organic solvents**	**Relative activity (%)**
Control	100 ± 1.13
Octane	175.48 ± 2.29
*n*-Hexane	256.78 ± 1.78
Cyclohexane	134.47 ± 3.74
*n*-Decane	189.45 ± 0.85
*n*-Heptane	279.46 ± 1.24
Toluene	218.6 ± 0.45
1-Octanol	114.78 ± 0.76
Petroleum ether	84.26 ± 1.39

*The enzyme activity of the non-organic solvent control was defined as 100%. The values shown are the average of triplicate determinations ± standard error (SE).*

**TABLE 4:** Effects of various organic solvents on recombinant BaPAD stability.

**Organic solvents**	**Concentration (%,v/v)**	**Relative activity (%)**	
		**1 h**	**12 h**
control	0	100 ± 0.14	100 ± 0.21
1-octanol	20	117.07 ± 0.94	79.75 ± 0.73
	30	96.50 ± 1.12	70.72 ± 1.02
	50	97.96 ± 0.39	57.01 ± 0.84
petroleum ether	20	104.39 ± 0.64	77.92 ± 0.94
	30	104.60 ± 0.42	85.22 ± 0.49
	50	105.38 ± 0.92	91.65 ± 0.61
Toluene	20	92.97 ± 2.77	60.20 ± 1.98
	30	103.95 ± 1.42	85.17 ± 0.83
	50	105.80 ± 1.79	85.78 ± 1.31
cyclohexane	20	106.18 ± 1.57	84.39 ± 1.13
	30	103.02 ± 0.39	81.56 ± 0.74
	50	106.58 ± 0.86	80.67 ± 0.58
n-hexane	20	89.13 ± 5.59	91.73 ± 2.12
	30	94.92 ± 0.47	85.11 ± 1.01
	50	90.74 ± 0.79	81.82 ± 0.36
n-decane	20	105.18 ± 1.75	87.55 ± 0.98
	30	92.79 ± 1.34	87.61 ± 1.89
	50	103.94 ± 1.29	91.05 ± 1.03
octane	20	96.01 ± 1.45	95.71 ± 0.95
	30	94.98 ± 1.75	89.77 ± 1.23
	50	100.41 ± 2.23	93.87 ± 2.03

*The values are the means of triplicate experiments with ± standard error (SE).*

### The Molecular Toxicity Analysis of BaPAD and Whole Cells

**FIGURE 3:**
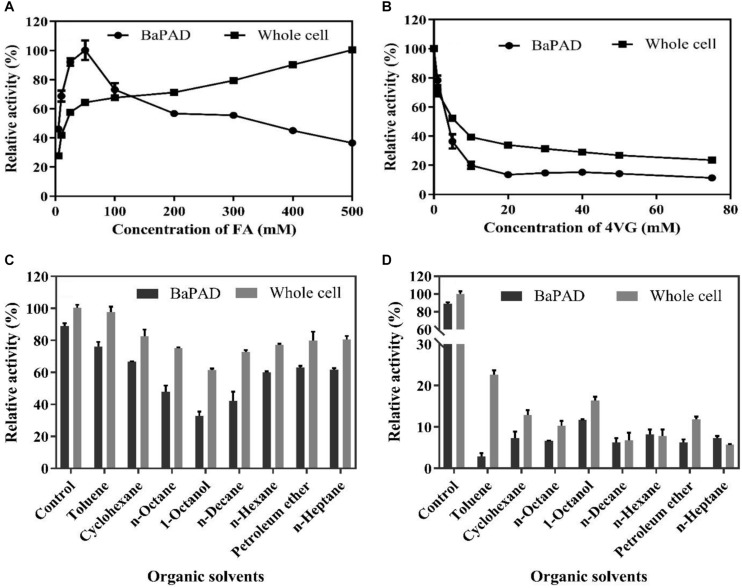
The inhibition of substrate, product, and organic solvents on the activities of BaPAD and whole cells. **(A)** Relative activities of the BaPAD and whole cells with different concentrations of FA. **(B)** Relative activities of the BaPAD and whole cells with different concentrations of 4-VG. **(C,D)** Relative activities of the BaPAD and whole cells in a pre-saturated buffer with different organic solvents and 10 g/L (66.7 mM) 4-VG or 100 g/L (666.7 mM) 4-VG, respectively.

The toxicities of FA and 4-VG on the activities of the BaPAD and whole cells were determined by measuring the relative activity in the presence of different concentrations of FA and 4-VG. As shown in Figure 3A, the BaPAD and whole cells exhibited rapid and steady activity increases as the FA concentration was increased from 1 to 50 mM. However, the activity of BaPAD declined sharply as the FA concentration exceeded 50 mM. In contrast, the activity of whole cells continued to increase slowly as the concentration of FA increased to 500 mM. These observations suggested that a high concentration of FAs has a significant inhibitory effect on the BaPAD enzyme activity but a lower toxicity to the whole cells. As shown in Figure 3B, with the increase of 4-VG, the catalytic activity of the BaPAD and whole cells decreased rapidly and subsequently tended to stabilize at 20% and 40% of the initial activity, respectively, indicating that 4-VG was highly toxic to the BaPAD and whole cells, even at low concentrations. Overall, these results clearly demonstrated that the intracellular enzymes in cells exhibited significantly higher resistances to the toxicities of FA and 4-VG than those of the free BaPAD, likely due to the additional protection of the cell membrane of *E. coli*.

The inhibitory effects on biocatalysts can be further strengthened when co-existing of 4-VG with the organic solvents during catalysis. Thus, the combined effects of the residual organic solvents and 4-VG dissolved in the aqueous phase on the activity of BaPAD and whole cell were analyzed by measuring the activity using the solvent and 4-VG pre-saturated buffer. As shown in Figure 3C, a strong inhibitory effect on the catalytic activities of the BaPAD and whole cells was observed when using the buffer pre-saturated with 66.7 mM of 4-VG and different organic solvents, in the following order: 1-octanol, *n*-decane, *n*-octane, *n*-hexane, cyclohexane, *n*-heptane, petroleum ether, and toluene. The relative catalytic activity of whole cell was reduced to 61% and 97%, respectively, in 66.7 mM of 4-VG and 1-octanol or toluene pre-saturated buffer compared to control. The inhibition became more serious when the buffer was pre-saturated with 666.7 mM of 4-VG and different organic solvents (Figure 3D). The relative catalytic activity of whole cell was reduced to 16 and 22%, respectively, in 666.7 mM of 4-VG and 1-octanol or toluene pre-saturated buffer compared to control, and the corresponding values were significantly reduced further for 666.7 mM of 4-VG and other organic solvent pre-saturated buffers. Comparatively, it could be concluded that the relative inhibition in 1-octanol and 666.7 mM of 4-VG pre-saturated buffer become weaker than that in the other organic solvents and 666.7 mM of 4-VG pre-saturated buffer (Figure 3D).

### Solvent Screening for Biphasic Reaction of Whole-Cell Catalysis

**FIGURE 4:**
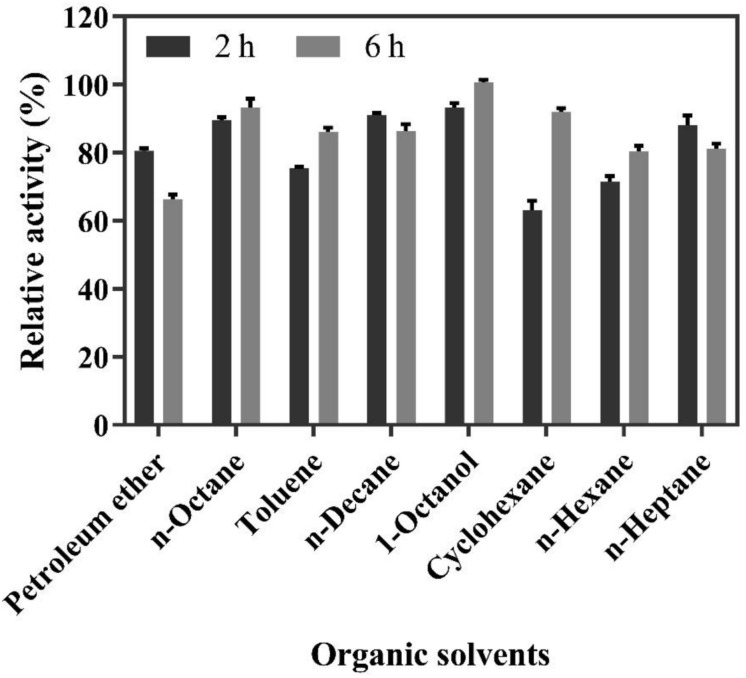
Relative activity of whole-cell catalysis for 2 and 6 h in the biphasic reaction system containing 200 mM FA and different organic solvents.

The solvent screening for the biphasic reaction was carried out in a biphasic system containing a Na_2_HPO_4_-citric acid buffer containing 200 mM of FA and different organic solvents for 2 and 6 h. As shown in Figure 4, the highest molar conversion yield (100%) of 4-VG was obtained in 1-octanol after 6 h of reaction, followed by *n*-octane, whose conversion yield reached up to 95%. Moreover, from safety, health, and environmental protection perspectives, 1-octanol is preferable to the other organic solvents tested (Grundtvig et al., 2018). Therefore, 1-octanol was selected as the organic solvent used in the subsequent experiments.

### 4-VG Bioproduction by Whole-Cell Catalysis in a 5 L Bioreactor

**FIGURE 5:**
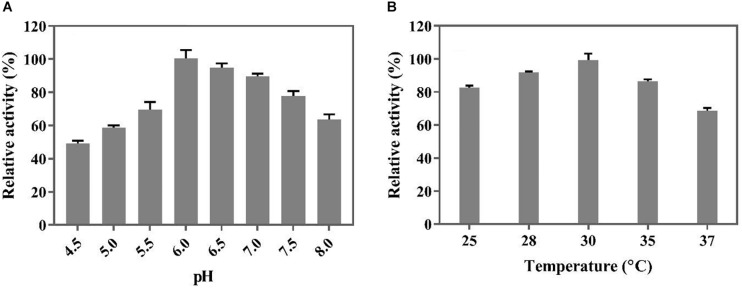
Optimization of conditions for whole cells catalysis of FA. **(A)** Effect of different pH on whole cells catalyzing FA; **(B)** Effect of different temperatures on whole cells catalyzing FA.

Before biotransformation in a 5 L bioreactor, the optimal temperature and pH for the whole-cell catalysis were determined in an organic solvent/aqueous biphasic system in shake flask cultures. As shown in Figure 5, the decarboxylation activity of the whole cells was the highest at a pH of 6.0 and temperature of 30°C. The optimal reaction temperature of the whole cells was lower than that of the free BaPAD (50°C), but the optimal pH was higher than that of the free BaPAD (pH 5.5).

**FIGURE 6:**
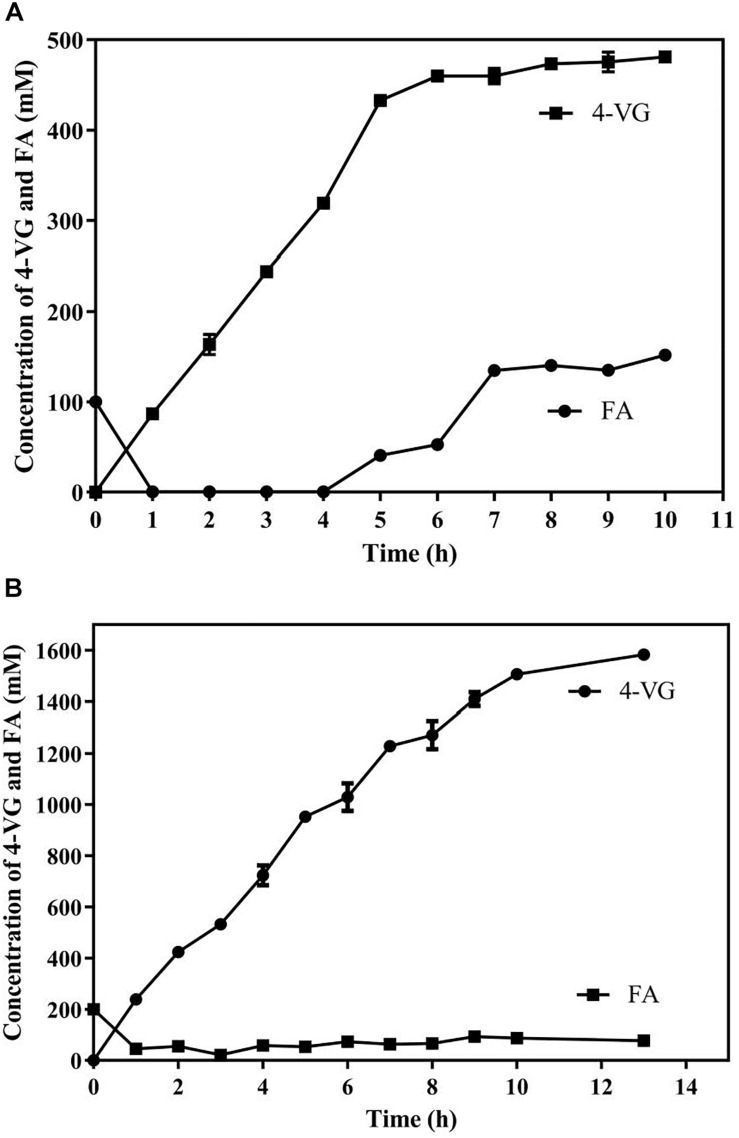
Bioproduction of 4-VG from ferulic acid by whole-cell catalysis in the **(A)** aqueous and **(B)** 1-octanol/aqueous biphasic systems.

Two systems, including an aqueous monophasic system and biphasic system with phase separation, with a substrate fed-batch strategy, were used and compared in terms of 4-VG yield and productivity (Figure 6). As shown in Figure 6A, in the aqueous system, the conversion rate of FA declined significantly after 5 h, and the amount of 4-VG accumulated slowly after 5 h. After 10 h of reaction, approximately 72 g/L (480 mM) of 4-VG was obtained from 650 mM FA, and the molar conversion yield and the productivity reached about 74% and 7.2 g/L/h, respectively. In the biphasic biological reaction system, as shown in Figure 6B, 4-VG accumulated rapidly at a higher rate for longer time compared to the aqueous system, and approximately 237.3 g/L (1580 mM) of 4-VG was obtained from 310 g/L (1598 mM) of FA in 13 h. In this reaction, the molar conversion yield and productivity reached 98.9% and 18.3 g/L/h, respectively.

## Discussion

In the current study, a new phenolic acid decarboxylase (BaPAD) was identified from *B. atrophaeus*. The substrate preference of BaPAD was similar to those of the PADs from *Bacillus* spp., such as *B. amyloliquefaciens, B. subtilis, B. pumilus*, and *B. licheniformis* (Degrassssi et al., 1995; Cavin et al., 1998; Jung et al., 2013; Hu et al., 2015), but different from that of the PAD from *Brettanomyces bruxellensis*, which had a greater activity toward FA than toward *p*-coumaric acid and caffeic acid (120:100:80) (Godoy et al., 2008).

Biocatalyst stability over broad ranges of the pH and high temperatures is necessary for industrial applications. The enzymatic properties of various PADs from different bacteria have been explored previously (Rodríguez et al., 2008; Landete et al., 2010; Gu et al., 2011; Jung et al., 2013). Most bacterial PADs exhibited activities at temperatures between 22 and 45°C and were susceptible to heat inactivation. For example, the highest activity of the PAD from *Bacillus* sp. BP-7 was found at pH 5.5 and 40°C, and remaining stable over a pH range from 5.0 to 9.0 when incubated for 1 h at room temperature (Prim et al., 2003). The PAD from *L. brevis* had an optimal activity at 22°C, but the PAD activity sharply decreased to only 12% of the maximal activity at 37°C (Landete et al., 2010). Recently, Ni et al. (2018) reported a thermal stable BcPad from *B. coagulans* DSM1, which showed an optimum pH and temperature at pH 6.0 and 50°C, respectively, and retained over 70% of the relative activity in a pH range of 5.0 to 7.0 and over 60% of its relative activity at 55°C. Compared to the previously characterized decarboxylases, the BaPAD obtained from *B. atrophaeus* was more stable over a wide range of conditions, especially at high temperatures. Furthermore, the *V*_*max*_ values of recombinant BaPAD toward p-coumaric, ferulic and caffeic acids was far higher than that from *B. licheniformis and B. subtilis*, suggesting that BaPAD might have a high potential in industrial applications (Cavin et al., 1998; Hu et al., 2015).

BaPAD possesses excellent catalytic activity and stability in various organic solvents even for 1-octanol. This organic solvent usually exhibits a strong inhibition on PADs (Jung et al., 2013; Hu et al., 2015). Generally, the use of enzymes in organic media is restricted because most enzymes are less active and stable in the presence of organic solvents due to their direct interactions with the essential water surrounding the enzymes (Ogino and Ishikawa, 2001; Doukyu and Ogino, 2010). Overall, the organic solvent tolerance of BaPAD was similar as that of BLPAD from *B. licheniformis* (Hu et al., 2015) but much higher than those of PADs from other bacteria, such as *B. amyloliquefaciens* (Jung et al., 2013) and *Mycobacterium colombiense* (Wuensch et al., 2013).

Scale bioproduction of 4-VG or 4-vinyl phenol (4-VP) from phenolic acids using PADs is generally hampered by low biocatalysis rates, yields, and product titers (Ben-Bassat et al., 2007; Jung et al., 2013). A number of efforts had been made to improve final product concentrations and production using biphasic systems. Up to 13.8 g/L (91.9 mM) of 4-VG (purity of 98.4%) were obtained from 25 g/L of FA by using recombinant *E. coli* cells overproducing *L. plantarum* PAD in the octane–aqueous two-phase system in a 2-L working volume bioreactor (Salgado et al., 2012b). Yang et al. (2009) reported that 21.3 g/L (141.9 mM) of 4-VG (95% conversion yield) was obtained by using *E. coli* harboring a FA decarboxylase from *B. pumilus* in 1L of an octane–aqueous two-phase system. These issues such as low yield and product concentrations are mainly caused by the toxicity of the substrate, product, and organic solvents and enzymatic inactivation (Tomek et al., 2015; Grundtvig et al., 2018). Although water-immiscible organic solvents were used in the biphasic system, trace solvents dissolved in the aqueous phase may have serious inhibitory effects on the biocatalysts (Doukyu and Ogino, 2010). So in this study, the inhibitory effects of individual FA and 4-VG, or combined effects of the residual organic solvents and 4-VG on the recombinant enzyme and whole cells of *E. coli* expressing BaPAD were comprehensively evaluated. It showed that FA or 4-VG exhibited more serious inhibitory effects on free BaPAD than whole cells of *E. coli* expressing BaPAD, indicating that the cell membrane has some protection on whole *E. coli* cells. The biotransformation employing whole cells harboring intracellular enzymes as biocatalysts has attracted much attention due to advantages over free enzyme systems, such as not requiring cell lysis and enzyme purification, which thereby significantly reduces the production cost (Wachtmeister and Rother, 2016). The higher tolerances of whole cells of *E. coli* expressing BaPAD than those of free BaPAD to FA and 4-VG may further make the whole cells to outperform the free BaPAD in the bioproduction of high titer 4-VG. 1-Octanol has a higher partition coefficient for 4-VG than other organic solvents (Jung et al., 2013; Hu et al., 2015). Thus, 4-VG could be efficiently extracted from the aqueous phase into the 1-octanol phase, and the residual concentration of 4-VG in the aqueous buffer could be still maintained at a very low level, even if the buffer was pre-saturated with high concentration of 4-VG (100 g/L) and 1-octanol. This attribute might be responsible for the relatively weaker toxicity of 4-VG to the BaPAD and whole cells compared to the other organic solvents in a pre-saturated buffer with 1-octanol and 100 g/L 4-VG (Figure 3D). Moreover, from safety, health, and environmental protection perspectives, 1-octanol is preferable to the other organic solvents tested (Grundtvig et al., 2018). So a biocompatible and highly efficient organic solvent/aqueous biphasic system was established using 1-octanol as the optimal organic phase for whole-cell catalysis. In this system, a large amount (310 g/L) of FA could be almost completely converted to 4-VG in a 5 L bioreactor in 13 h, which are the highest values reported to date. This study clearly demonstrated that this biocatalysis method may be competitive with chemical syntheses using metal catalysts under harsh conditions.

## Conclusion

In this study, a new phenolic acid decarboxylase (BaPAD) from *Bacillus atrophaeus* was characterized and whole *Escherichia coli* cells harboring intracellular BaPAD was used for 4-VG bioproduction. A high concentration 4-vinylguaiacol was produced by using whole-cell catalysis in a biocompatible and highly efficient biphasic system, which may be attributed to the better properties of the new phenolic acid decarboxylase BaPAD, such as the good tolerance to various organic solvents, high thermostability, and excellent catalytic capability in the biphasic system compared to the previous characterized PADs.

## Data Availability

Publicly available datasets were analyzed in this study. The data can be found here: https://www.ncbi.nlm.nih.gov/search/all/?term=AKL86192.1 and https://www.ncbi.nlm.nih.gov/nuccore/MN011580.

## Author Contributions

LuL contributed to the study design, performed the bulk of the experimental work, analyzed and interpreted the data, and drafted the manuscript. LiL and SD contributed to the study design, analyzed and interpreted the data, and revised the manuscript. All authors read and approved the final manuscript.

## Conflict of Interest Statement

The authors declare that the research was conducted in the absence of any commercial or financial relationships that could be construed as a potential conflict of interest.
