# Supplementary material for: Bioproduction of High-Concentration 4-Vinylguaiacol Using Whole-Cell Catalysis Harboring an Organic Solvent-Tolerant Phenolic Acid Decarboxylase From Bacillus atrophaeus
Source: Front Microbiol. 2019 Aug 6;10:1798. doi: 10.3389/fmicb.2019.01798 (PMC6691155; doi:10.3389/fmicb.2019.01798)
Supplement: Supplementary file 1 [file Table_1.DOCX]

**Supplementary Information**

**Bioproduction of high concentration 4-vinylguaiacol using whole cell catalysis harboring an organic solvent-tolerant phenolic acid decarboxylase from *Bacillus atrophaeus***

Lulu Li^1^; Liangkun Long^1^, Shaojun Ding^1^*

The Co-Innovation Center of Efficient Processing and Utilization of Forest Resources, Jiangsu Key Lab for the Chemistry & Utilization of Agricultural and Forest Biomass, College of Chemical Engineering, Nanjing Forestry University, Nanjing, 210037, China

*Corresponding author: Dr. Shaojun Ding

Tel: +86 25 85427543

Fax: +86 25 85418873

E-mail: dshaojun@hotmail.com

Table S1 Effect of various metal ions and chemical reagents on BaPAD activity.

| Metal ions and chemical reagents | Relative activity (%) | |
| --- | --- | --- |
|  | 5 mM | 10 mM |
| Cu^2+^ | 95.68±0.61 | 49.24±0.61 |
| Li^+^ | 92.99±1.67 | 89.03±1.99 |
| Zn^2+^ | 95.60±1.78 | 91.89±1.27 |
| Ni^+^ | 97.40±0.78 | 96.46±0.07 |
| Mg^2+^ | 96.74±0.21 | 96.42±2.36 |
| Mn^2+^ | 97.61±0.25 | 95.30±2.1 |
| Ca^2+^ | 99.51±0.34 | 98.39±2.01 |
| Co^2+^ | 101.18±2.11 | 90.58±3.05 |
| Fe^3+^ | 102.03±1.86 | 90.02±0.43 |
| K^+^ | 94.65±1.42 | 76.59±1.17 |
| NH^4+^ | 88.42±1.24 | 76.76±0.2 |
| EDTA | 80.78±1.16 | 72.37±2.65 |
| TritonX-100 | 112.39±0.6 | 90.68±2.27 |
| SDS | ND | ND |
| Control | 100±0.25 | 100±1.16 |

The enzyme activity of the non-metal ion-containing control was defined as 100%. Values shown were the average of triplicate determinations ± standard error (SE).

ND not detected.


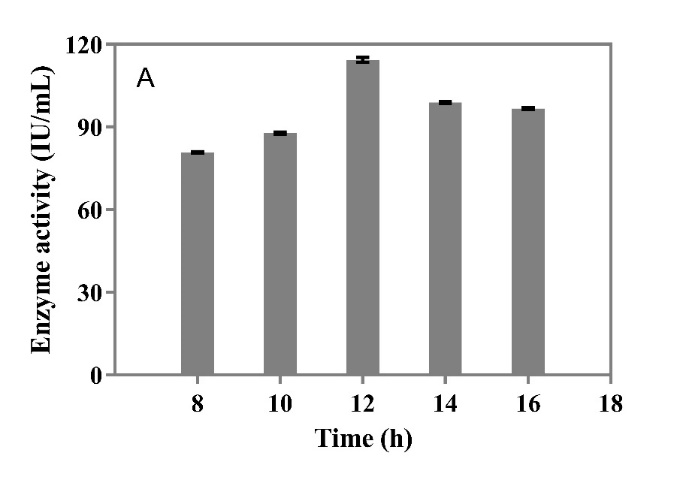

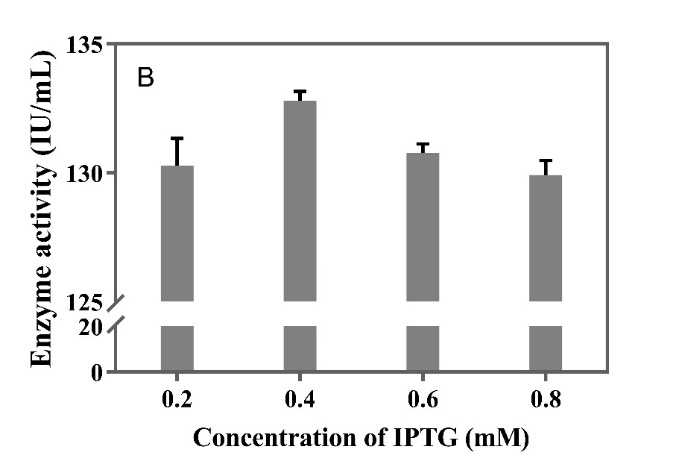

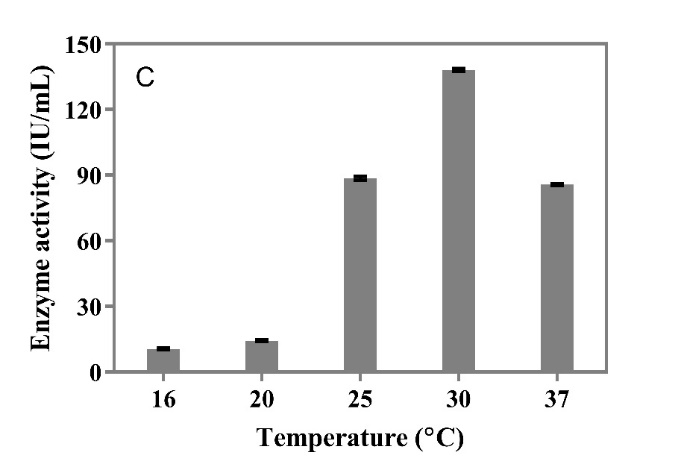


Figure S1. Effect of induction time (A), IPTG concentration (B) and temperature (C) on enzyme productions. Values shown are means of triplicate determinations ± standard error (SE).
